# Oral small-molecule GLP-1 receptor agonists: a new Frontier in cardiometabolic medicine

**DOI:** 10.3389/fphar.2026.1933018

**Published:** 2026-08-28

**Authors:** Jinkai Guo, Hua Chen

**Affiliations:** 1 Baotou Medical College, Baotou, Inner Mongolia, China; 2 Department of Cardiology, Inner Mongolia Autonomous Region People’s Hospital, Hohhot, China

**Keywords:** cardiovascular outcomes, danuglipron, nonpeptide, obesity, oral GLP-1 receptor agonist, orforglipron, small molecule, type 2 diabetes

## Abstract

Glucagon-like peptide-1 receptor agonists (GLP-1 RAs) have revolutionized the management of type 2 diabetes (T2D) and obesity, with injectable agents demonstrating significant cardiovascular (CV) risk reduction in pivotal trials including LEADER, SELECT, and SUMMIT. However, the reliance on injectable peptide formulations has constrained global accessibility and long-term adherence. The recent emergence of orally bioavailable, nonpeptide, small-molecule GLP-1 RAs—exemplified by orforglipron (Foundayo), danuglipron, and other investigational candidates—marks a paradigm shift in incretin-based therapy. Unlike oral semaglutide, which delivers a peptide via sodium N-[8-(2-hydroxybenzoyl) amino] caprylate (SNAC)-mediated absorption with stringent dosing requirements, small-molecule GLP-1 RAs evade enzymatic degradation and facilitate once-daily oral dosing without fasting restrictions or cold-chain logistics. This review synthesizes the pharmacology, Phase 3 clinical evidence (including the ACHIEVE and ATTAIN programs), comparative efficacy against injectable GLP-1 RAs and SGLT2 inhibitors, safety data from over 11,000 patients, and the evolving regulatory landscape—including FDA approval of orforglipron (Foundayo) for chronic weight management in April 2026. We explore positioning within the cardiometabolic treatment algorithm, emphasize limits of indirect comparisons, identify the unresolved cardiovascular outcomes question linked to biased partial agonism, and discuss implications for global access.

## Highlights

Question: Can oral small-molecule, nonpeptide GLP-1 receptor agonists deliver glycemic control and weight loss comparable to injectable GLP-1 RAs and oral semaglutide, while eliminating injection and fasting barriers?

Findings: The Phase 3 ACHIEVE program demonstrates that orforglipron is non-inferior to dapagliflozin for HbA1c reduction (ACHIEVE-2) and effective as add-on to insulin glargine (ACHIEVE-5), with pooled hepatic safety data from 11,220 participants. A population-adjusted *indirect* treatment comparison estimated greater percentage weight loss with oral semaglutide 25 mg than orforglipron 36 mg (mean difference −3.2 percentage points), but this is not head-to-head evidence and should not be read as definitive comparative ranking. Orforglipron offers dosing convenience (no fasting requirement; no cold chain).

Meaning: Oral small-molecule GLP-1 RAs expand the incretin therapeutic landscape by prioritizing accessibility. Their role in cardiovascular prevention remains unresolved until dedicated cardiovascular outcomes evidence mature—including post-marketing commitments now attached to regulatory approval for weight management.

## 1 Introduction

Glucagon-like peptide-1 (GLP-1) is an incretin hormone secreted primarily by intestinal L-cells in response to nutrient ingestion. Physiologically, GLP-1 augments glucose-dependent insulin secretion, suppresses glucagon, slows gastric emptying, and reduces appetite through central and peripheral pathways—actions that collectively lower postprandial glycemia and energy intake without the hypoglycemia risk typical of insulin secretagogues that act independently of glucose (Zhang et al., 2017). These properties provide a clear therapeutic rationale for GLP-1 receptor agonism in type 2 diabetes (T2D) and obesity: restoring or amplifying an endogenous enteroinsular and satiety axis that is impaired or insufficient in cardiometabolic disease.

The past decade has witnessed an unprecedented expansion in the therapeutic armamentarium built on this biology. Injectable GLP-1 receptor agonists (GLP-1 RAs) have progressed from second-line glucose-lowering agents to cornerstone therapies with proven cardiovascular and renal benefits across T2D, obesity, and heart failure with preserved ejection fraction (HFpEF) (Badve et al., 2025; Marso et al., 2016a; Marso et al., 2016b; Nong et al., 2025). The SELECT trial demonstrated that semaglutide 2.4 mg reduced major adverse cardiovascular events (MACE) by 20% in adults with established cardiovascular disease and overweight or obesity without diabetes (Lincoff et al., 2023). The SUMMIT trial extended these findings, showing that tirzepatide reduced the composite of cardiovascular death or worsening heart failure events in patients with HFpEF and obesity (Packer et al., 2025). Simultaneously, the SOUL trial confirmed that oral semaglutide—a peptide formulation co-formulated with the absorption enhancer sodium N-[8-(2-hydroxybenzoyl) amino] caprylate (SNAC)—reduced MACE by 14% (hazard ratio 0.86) in high-risk adults with T2D (McGuire et al., 2025; Pop-Busui et al., 2026).

Yet despite these achievements, significant barriers persist. Injectable GLP-1 RAs require cold-chain storage, subcutaneous self-administration, and dose-escalation schedules that contribute to substantial real-world discontinuation within the first year of therapy (Kansakar et al., 2026). Oral semaglutide, while eliminating the need for injection, demands strict fasting before and after administration, limiting its practical convenience. These constraints have motivated an intensive search for orally bioavailable GLP-1 RAs that are free from peptide-related limitations.

Two fundamentally distinct strategies have emerged. The first—oral delivery of peptide agonists using permeation enhancers—is exemplified by oral semaglutide (Rybelsus) and oral semaglutide for weight management (Wegovy tablets, FDA approval December 2025) (FDA NDA 218316., 2025). The second, more radical approach involves true small-molecule, nonpeptide GLP-1 receptor agonists designed *de novo* for oral bioavailability without peptide constraints. Orforglipron (LY3502970; Eli Lilly; Foundayo), danuglipron (PF-06882961; Pfizer), and the discontinued lotiglipron (PF-07081532) represent the vanguard of this new class (Son et al., 2026).

The pharmacological rationale for small-molecule GLP-1 RAs is compelling. By engaging the GLP-1 receptor through a binding mode distinct from peptide agonists, these agents achieve biased signaling profiles that may confer differential efficacy and tolerability (Patel, 2026). Their resistance to proteolytic degradation enables consistent oral pharmacokinetics without absorption enhancers, fasting requirements, or cold-chain distribution—features that could expand global access to incretin-based therapy, particularly in low-resource settings.

**FIGURE 1:**
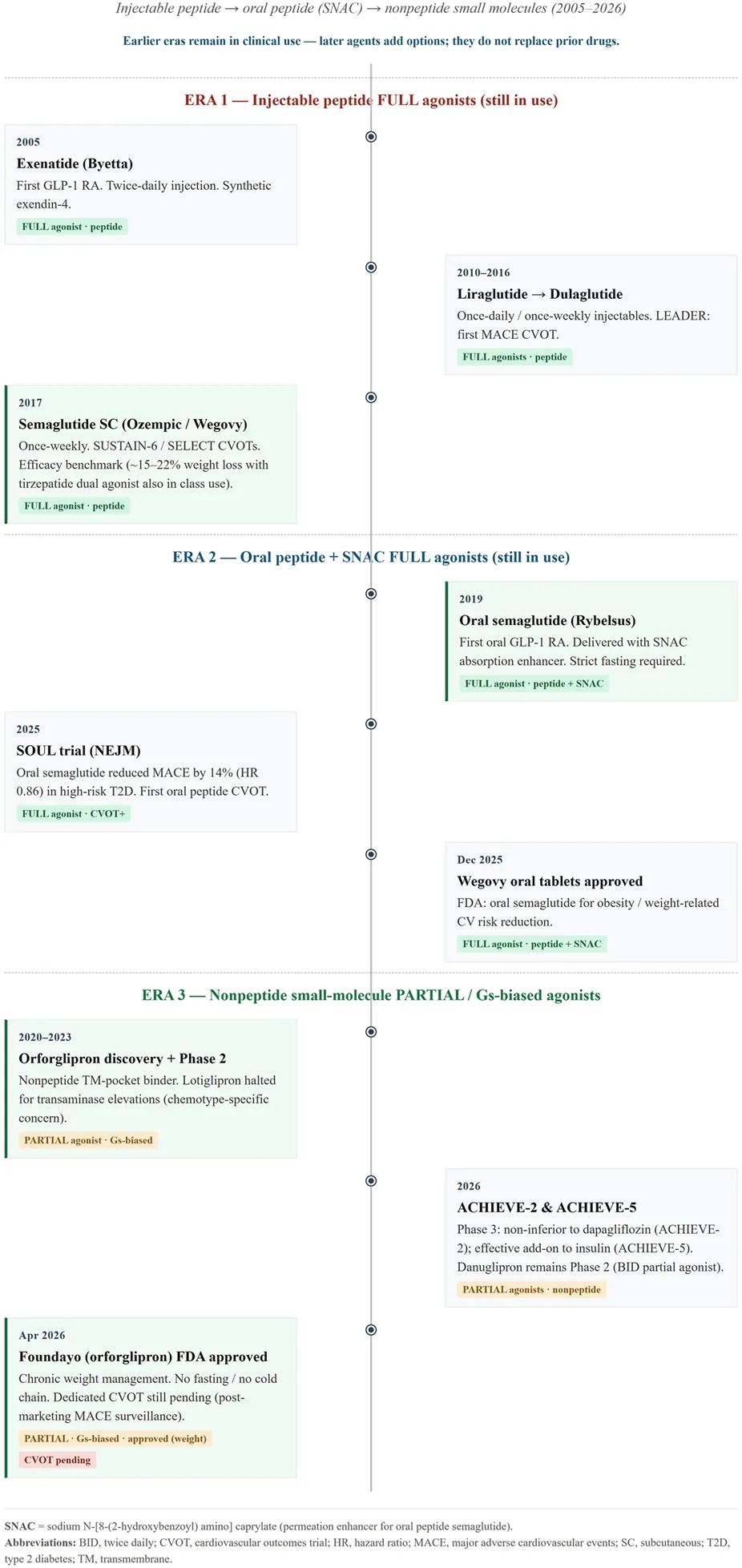
Evolution of oral GLP-1 receptor agonist therapy. Timeline across three eras that remain clinically coexistent (earlier agents are not replaced by later ones). Era 1 injectable peptide full agonists (exenatide, liraglutide, dulaglutide, semaglutide injectable); Era 2 oral peptide full agonists delivered with SNAC (sodium N-[8-(2-hydroxybenzoyl) amino] caprylate) absorption enhancement (oral semaglutide); Era 3 nonpeptide small-molecule partial agonists with biased Gs signaling (orforglipron; danuglipron investigational; lotiglipron discontinued). Key milestones include SOUL (oral semaglutide MACE ↓14%), Wegovy oral approval (Dec 2025), ACHIEVE Phase 3 publications (2026), and Foundayo (orforglipron) FDA approval for weight management (Apr 2026). Abbreviations: ASCVD, atherosclerotic cardiovascular disease; CKD, chronic kidney disease; CVOT, cardiovascular outcomes trial; MACE, major adverse cardiovascular events; NDA, New Drug Application; SNAC, sodium N-[8-(2-hydroxybenzoyl) amino] caprylate; T2D, type 2 diabetes.

This narrative review synthesizes the pharmacological basis, clinical evidence, comparative positioning, regulatory trajectory, and future directions of oral small-molecule GLP-1 RAs, with emphasis on cardiovascular prevention implications. The evolution from injectable peptide GLP-1 RAs to oral small-molecule agents is illustrated in Figure 1; a three-era comparative summary is shown in Table 1.

**TABLE 1:** Pharmacology and therapeutic efficacy across three coexistent eras. Same GLP-1 receptor. Different molecular engagement. Not a head-to-head ranking.

Domain	Era 1 Injectable peptide	Era 2 Oral peptide + SNAC	Era 3 Nonpeptide small molecule
Agonism	FULL agonist	FULL agonist	PARTIAL/Gs-biased
Exemplar drugs	Exenatide, liraglutide, dulaglutide, semaglutide SC, tirzepatide (dual GIP/GLP-1)	Oral semaglutide (Rybelsus; Wegovy oral)	Orforglipron (Foundayo); danuglipron (Phase 2); lotiglipron (discontinued)
Pharmacology	Classical peptide two-domain GLP-1R binding	Same peptide pharmacology + SNAC absorption enhancer	Transmembrane-pocket nonpeptide binding—same receptor, different engagement
Therapeutic efficacy	Weight loss ∼15%–22% CVOT: LEADER/SELECT/SUMMIT	Weight loss ∼15% (25 mg) CVOT: SOUL MACE ↓14%	Weight loss ∼8%–10% (36 mg) Glycemia: ACHIEVE-2/5 CVOT: pending
Practical constraints	Injection + cold chain	Strict fasting; no cold chain	No fasting; no cold chain
Clinical use today	REMAINS standard of care	REMAINS in use	ADDS option—does not replace Eras 1–2

SNAC = sodium N-[8-(2-hydroxybenzoyl) amino] caprylate. Weight-loss ranges are approximate cross-trial estimates. Abbreviations: CVOT, cardiovascular outcomes trial; MACE, major adverse cardiovascular events; SC, subcutaneous.

Side-by-side comparison of injectable peptide full agonists, SNAC-enhanced oral peptide full agonists, and nonpeptide small-molecule partial agonists (biased Gs signaling), including representative weight-loss ranges, CVOT status, and practical constraints. Prior eras remain in clinical use after introduction of small-molecule agents.

### 1.1 Literature search and scope

We conducted a narrative (not systematic) literature review. PubMed/MEDLINE, ClinicalTrials.gov, and publicly available FDA regulatory documents were searched from inception through July 2026 using combinations of the terms *orforglipron*, *danuglipron*, *lotiglipron*, *oral GLP-1*, *small-molecule GLP-1 receptor agonist*, *nonpeptide GLP-1*, *ACHIEVE*, and *ATTAIN*. We prioritized Phase 2/3 randomized trials, pooled safety analyses, meta-analyses, mechanistic structural studies, and regulatory milestones. Secondary analyses and narrative reviews were included when they clarified cardiovascular, hepatic, or comparative positioning questions. Preclinical chemistry reports were cited selectively to illustrate pipeline diversity. Because this is a narrative review, we did not register a protocol or apply formal PRISMA screening metrics; study selection favored peer-reviewed primary sources and avoided unpublished datasets.

## 2 Pharmacological basis of small-molecule GLP-1 receptor agonists

### 2.1 Peptide vs. nonpeptide agonism at the GLP-1 receptor

The GLP-1 receptor is a class B G protein-coupled receptor (GPCR) with a large extracellular domain that binds the C-terminal helix of endogenous GLP-1, facilitating N-terminal engagement with the transmembrane domain to trigger Gαs-mediated cyclic AMP (cAMP) signaling (Zhang et al., 2017). Injectable peptide GLP-1 RAs (semaglutide, liraglutide, dulaglutide) and the dual GIP/GLP-1 agonist tirzepatide are large polypeptides (3–5 kDa) that mimic this two-domain binding mechanism, requiring parenteral administration due to negligible oral bioavailability.

Small-molecule GLP-1 RAs adopt a fundamentally different binding mode while still activating the same GLP-1 receptor. Crystallographic and cryo-electron microscopy studies reveal that orforglipron and related nonpeptide agonists bind within the transmembrane orthosteric pocket, engaging residues distinct from those contacted by peptide agonists (Zhao et al., 2020; Kawai et al., 2020). This binding produces partial agonism with signal bias: robust Gαs–cAMP activation, but attenuated β-arrestin recruitment relative to peptide full agonists (Jones et al., 2018). Importantly, “same receptor, different engagement” means shared downstream glycemic and satiety pathways are expected, yet receptor trafficking, desensitization, and tissue-level effector coupling may diverge. The functional consequences of biased agonism for long-term cardiovascular protection remain unknown: peptide GLP-1 RAs with completed CVOTs are predominantly full (or dual-incretin) agonists whose cardioprotection may depend on Gs-dependent metabolic effects, weight loss, anti-inflammatory actions, and possibly β-arrestin–linked pathways that small-molecule partial agonists engage less strongly. Until event-driven CVOTs (or adequate post-marketing cardiovascular programs) mature, biomarker improvements and glycemic/weight efficacy cannot be extrapolated to MACE equivalence.

### 2.2 Orforglipron (LY3502970)

Orforglipron is the most clinically advanced oral small-molecule GLP-1 RA. It is a partial agonist at the human GLP-1 receptor (EC50 ∼2 nM for cAMP) with >1,000-fold selectivity over related class B GPCRs (Kawai et al., 2020). Preclinical studies demonstrated central nervous system penetration, engagement of hypothalamic GLP-1 receptors, and suppression of food intake in rodent models—findings consistent with the weight loss observed in clinical trials.

Phase 1 studies established once-daily oral dosing without food restrictions as feasible, with a half-life supporting 24-h receptor coverage (Frias et al., 2023). Unlike oral semaglutide, which requires administration on an empty stomach with no more than 120 mL of water followed by a ≥30-min fasting period, orforglipron can be taken without regard to meals—a distinction likely to translate into meaningful adherence advantages. A dedicated drug-drug interaction study confirmed no clinically relevant effects on the pharmacokinetics of common cardiovascular medications, including atorvastatin, metformin, and warfarin (Morse et al., 2026).

### 2.3 Danuglipron and the Pfizer pipeline

Danuglipron (PF-06882961) is a structurally distinct small-molecule GLP-1 RA developed by Pfizer. Early clinical development included a Phase 1 multiple ascending-dose study (Saxena et al., 2021), and subsequent randomized evidence synthesized in meta-analysis shows HbA1c reductions (mean difference vs. placebo approximately −0.90%; 95% CI −1.06 to −0.74) and weight loss (approximately −2.17 kg; 95% CI −3.10 to −1.23) in patients with T2D (Zhou et al., 2025). Danuglipron requires twice-daily dosing, and gastrointestinal adverse events have been a prominent tolerability consideration in the published program (Zhou et al., 2025; Saxena et al., 2021).

Lotiglipron (PF-07081532), a once-daily follow-on candidate with improved pharmacokinetics, was discontinued in 2023 after Phase 1 studies revealed transaminase elevations (Wharton et al., 2026). This experience underscores a class-relevant lesson: small-molecule GLP-1 RAs share a therapeutic target but not a uniform hepatic risk profile. Proposed contributors to lotiglipron-associated enzyme elevations include chemotype-specific metabolic liabilities (reactive metabolite formation and/or off-target hepatocyte stress) rather than on-target GLP-1 receptor activation per se—because peptide GLP-1 RAs and orforglipron have not reproduced a Hy’s Law signal in large development programs, and orforglipron’s pooled Phase 3 hepatic data show mean ALT/AST *declines* consistent with weight-loss–related improvement in steatosis (Wharton et al., 2026). Definitive molecular toxicology linking lotiglipron’s scaffold to idiosyncratic DILI has not been fully disclosed in the peer-reviewed literature; accordingly, hepatic risk should be framed as molecule-specific pending structure–toxicity disclosure, not as an inevitable property of nonpeptide GLP-1 agonism. Pfizer subsequently redirected resources toward next-generation candidates designed to mitigate hepatotoxicity while preserving potency.

### 2.4 Emerging small-molecule candidates

The success of orforglipron has catalyzed a wave of medicinal chemistry efforts. Fragment-based and structure-guided design approaches have yielded novel chemotypes, including 1H-benzo [d]imidazole-based agonists and dual GLP-1R/GIPR small-molecule scaffolds (Galiano and Notario-Pérez, 2026; Li et al., 2026). Several academic and industry groups have reported orally bioavailable compounds with picomolar potency in preclinical models, suggesting that a pipeline of structurally diverse small-molecule incretin mimetics is on the horizon.

## 3 Clinical evidence

### 3.1 Glycemic control: the ACHIEVE program

The ACHIEVE Phase 3 program represents the most comprehensive evaluation of an oral small-molecule GLP-1 RA to date. ACHIEVE-2, a 40-week, multicenter, randomized, open-label, non-inferiority trial, compared three doses of once-daily orforglipron (3 mg, 12 mg, and 36 mg) against dapagliflozin 10 mg in adults with T2D inadequately controlled on metformin monotherapy (HbA1c 7.0%–10.5%, BMI ≥23 kg/m^2^); 962 participants were randomized (Welch et al., 2026). Using a non-inferiority margin of 0.3% for the primary endpoint of HbA1c change from baseline at week 40 (treatment-regimen estimand), all orforglipron doses were non-inferior to dapagliflozin, with larger point estimates of HbA1c reduction at higher doses (mean changes approximately −1.23%, −1.50%, and −1.56% with 3, 12, and 36 mg vs. −0.81% with dapagliflozin). Weight loss was greater with orforglipron across dose levels, consistent with GLP-1 receptor–mediated effects on satiety and gastric emptying. Gastrointestinal events were the most frequent adverse events, and treatment discontinuations were more common with orforglipron than with dapagliflozin (Welch et al., 2026).

ACHIEVE-5 evaluated orforglipron (3, 12, and 36 mg) vs. placebo as add-on therapy to titrated insulin glargine in 546 adults with T2D and inadequate glycemic control (median age 61 years; 52.9% male; median T2D duration 14.6 years) (Giorgino et al., 2026). At week 40, mean HbA1c changes were approximately −1.58%, −1.88%, and −1.82% with orforglipron 3, 12, and 36 mg, respectively, vs. −0.79% with placebo; each dose was superior to placebo for HbA1c reduction. Mean percentage body-weight change was approximately −2.6%, −4.8%, and −5.4% vs. +0.2% with placebo (Giorgino et al., 2026).

Complementing the ACHIEVE program, the ATTAIN series evaluated orforglipron in obesity without diabetes. ATTAIN-1 demonstrated clinically meaningful weight loss at 40 weeks with an oral nonpeptide GLP-1 RA, expanding options beyond injectable incretin therapies (Zaman and Amin, 2026). Exact percentage weight-loss estimates should be taken from the primary ATTAIN publications and are summarized al caveats in Tables 1, 2.

**TABLE 2:** Pharmacological and clinical comparison of oral GLP-1 RA strategies. All agents activate GLP-1R — but peptide FULL agonism vs nonpeptide PARTIAL / biased engagement differs.

Property	Injectable peptide (semaglutide/tirzepatide)	Oral peptide (oral semaglutide)	Orforglipron (Foundayo)	Danuglipron
Receptor target	Same target: GLP-1 receptor (GLP-1R)			
Molecular engagement	Peptide two-domain binding	Peptide + SNAC enhancer	Nonpeptide TM-pocket binder	Nonpeptide TM-pocket binder
Agonism/bias	Full agonist	Full agonist	Partial, Gs-biased	Partial agonist
Administration	SC injection (typically weekly)	Oral tablet, daily	Oral tablet, daily	Oral tablet, twice daily
Fasting/cold chain	No fasting; cold chain required	Strict fasting; no cold chain	No fasting; no cold chain	No fasting; no cold chain
Approx. weight loss*	∼15%–22%	∼15% (25 mg)	∼8%–10% (36 mg)	∼2%–3% (Phase 2)
CVOT/MACE	Completed (multiple)	Completed (SOUL ↓14%)	Not completed	Not completed
Hepatic notes	Class-typical	Class-typical	Pooled Phase 3 reassuring; post-marketing DILI watch	Acceptable in Phase 2; less mature
US regulatory (mid-2026)	Approved	Approved	Approved for weight management (April 2026); T2D labeling maturing	Phase 2

* Cross-trial estimates — not head-to-head. SNAC, sodium N-[8-(2-hydroxybenzoyl) amino] caprylate; BID implied by twice-daily dosing; DILI, drug-induced liver injury; TM, transmembrane; CVOT, cardiovascular outcomes trial; MACE, major adverse cardiovascular events.

A 2025 systematic review and meta-analysis by Zhou et al. synthesized data from eight randomized controlled trials (1,454 participants) comparing danuglipron and orforglipron against placebo (Zhou et al., 2025). Orforglipron reduced HbA1c by −1.02% (95% CI: −1.18 to −0.86), fasting plasma glucose by −26.9 mg/dL (95% CI: −31.1 to −22.8), and body weight by −6.28 kg (95% CI: −8.45 to −4.11). Danuglipron produced more modest effects (HbA1c −0.90%, weight −2.17 kg), likely reflecting its twice-daily dosing. Both agents were associated with increased gastrointestinal adverse events compared with placebo.

A separate cardiometabolic-focused meta-analysis by Alper and colleagues reported improvements in systolic blood pressure, triglycerides, and waist circumference with orforglipron, while noting that dedicated cardiovascular outcomes data remain absent (Alper et al., 2026).

### 3.2 Weight loss: comparative efficacy (indirect evidence only)

No head-to-head randomized trial has compared orforglipron with oral or injectable semaglutide at matched obesity doses. A population-adjusted indirect treatment comparison (ITC) using individual patient data from OASIS 4 (oral semaglutide 25 mg) and aggregate data from ATTAIN-1 (orforglipron 36 mg) in adults with overweight or obesity without diabetes estimated a greater percentage body-weight reduction with oral semaglutide 25 mg (mean difference −3.2 percentage points; 95% CI −5.9 to −0.4) (Michalak et al., 2026). Discontinuation due to any adverse event was higher with orforglipron in that ITC model (odds ratio 4.1; 95% CI 1.3–13.0), driven largely by gastrointestinal events. These estimates are hypothesis-generating: residual confounding from differing trial populations, background therapy, titration schedules, and estimands cannot be excluded. We therefore avoid ranking agents by “superiority” on the basis of ITC alone; clinical choice should weigh convenience, tolerability, regulatory indication, and (where available) cardiovascular evidence.

### 3.3 Cardiovascular and metabolic biomarkers

Phase 3 data have demonstrated favorable effects of orforglipron on cardiovascular risk surrogates including systolic blood pressure, diastolic blood pressure, and triglyceride levels (Alper et al., 2026). High-sensitivity C-reactive protein reductions have been observed, consistent with anti-inflammatory effects described for peptide GLP-1 RAs. However, biomarker improvements do not substitute for dedicated cardiovascular outcomes trials. The SOUL trial established that oral semaglutide reduces MACE by 14% (McGuire et al., 2025); whether orforglipron—as a Gs-biased partial agonist—confers equivalent cardiovascular protection remains unproven and is a central translational question linking pharmacology to outcomes (see Section 6.1).

### 3.4 Safety and tolerability

The hepatic safety of orforglipron has been scrutinized precisely because lotiglipron’s transaminase signal raised concern that small-molecule GLP-1 RAs might share a chemotype-linked hepatotoxic liability. A pooled analysis of 11,220 participants across seven Phase 3 trials (orforglipron n = 6,920; pooled comparator n = 4,300) with follow-up to 104 weeks provides the strongest available clinical reassurance for orforglipron specifically (Wharton et al., 2026). Mean ALT and AST decreased from baseline in orforglipron-treated participants, consistent with improvements in hepatic steatosis from weight loss. Categorical elevations were balanced between groups. Six participants (0.1%) in each group had ALT or AST ≥3× ULN with concurrent total bilirubin ≥2× ULN; alternative etiologies accounted for orforglipron cases in the published analysis, and no case met Hy’s Law criteria for drug-induced liver injury. Mechanistically, the dissociation between lotiglipron’s early enzyme signal and orforglipron’s pooled profile supports molecule-specific metabolic risk rather than on-target GLP-1R toxicity; nonetheless, enhanced post-marketing pharmacovigilance for DILI remains appropriate for a first-in-class nonpeptide agonist.

Gastrointestinal adverse events—predominantly nausea, vomiting, diarrhea, and constipation—represent the most common tolerability limitation. Dose-escalation strategies (initiating at 3 mg and titrating to 12 or 36 mg over 4–8 weeks) mitigate GI intolerability. The development program has not identified safety signals related to medullary thyroid carcinoma, acute pancreatitis, or severe hypoglycemia (when used without insulin secretagogues).

An exploratory study in patients with moderate-to-severe obstructive sleep apnea (OSA) and obesity (ATTAIN-OSA) is evaluating orforglipron for improvements in apnea-hypopnea index, consistent with weight-loss-mediated amelioration of OSA severity (Malhotra et al., 2026).

## 4 Comparative positioning

### 4.1 Oral small-molecule vs. oral peptide GLP-1 RAs

The coexistence of two mechanistically distinct oral GLP-1 RA strategies—SNAC-enhanced peptide delivery (oral semaglutide) and direct small-molecule agonism (orforglipron)—creates a nuanced therapeutic landscape. Oral semaglutide 14 mg (Rybelsus) has established cardiovascular outcomes data through the SOUL trial (MACE reduction 14%), a body of evidence that orforglipron currently lacks as a completed dedicated CVOT (McGuire et al., 2025). Oral semaglutide 25 mg (Wegovy tablets) extends oral peptide therapy to weight management (FDA NDA 218316, 2025). The ITC estimating greater weight loss with oral semaglutide 25 mg vs. orforglipron 36 mg (Michalak et al., 2026) is informative but not definitive; convenience and fasting flexibility may still favor the small molecule for selected patients.

Key differentiating features:

- Dosing convenience: orforglipron can be taken without regard to meals; oral semaglutide requires strict fasting conditions.
- Cold chain: neither requires refrigeration.
- Evidence maturity: oral semaglutide has a completed CVOT (SOUL); orforglipron’s long-term cardiovascular program is still maturing despite weight-management approval.
- Tolerability: cross-trial comparisons suggest more GI discontinuations with orforglipron in some ITC models, but head-to-head trials are lacking.
- Agonism: peptide agents behave as full agonists at GLP-1R; orforglipron and danuglipron are nonpeptide partial agonists with biased Gs signaling (Table 2).

### 4.2 Oral small-molecule vs. injectable GLP-1 RAs

Injectable GLP-1 RAs—particularly semaglutide 2.4 mg (Wegovy) and tirzepatide (Mounjaro/Zepbound)—set the current efficacy benchmark for weight loss (15%–22% body weight reduction) and have established cardiovascular benefit through dedicated outcomes trials (Marso et al., 2016b; Lincoff et al., 2023; Packer et al., 2025). Oral small-molecule GLP-1 RAs are unlikely to surpass these agents on peak efficacy. Their value proposition resides in accessibility: elimination of injection barriers, cold-chain independence, and potentially lower manufacturing costs. For patients who decline or discontinue injectable therapy, oral small-molecule GLP-1 RAs may represent a clinically meaningful alternative that preserves a substantial fraction of injectable efficacy.

### 4.3 Oral small-molecule GLP-1 RA vs. SGLT2 inhibitors

**FIGURE 2:**
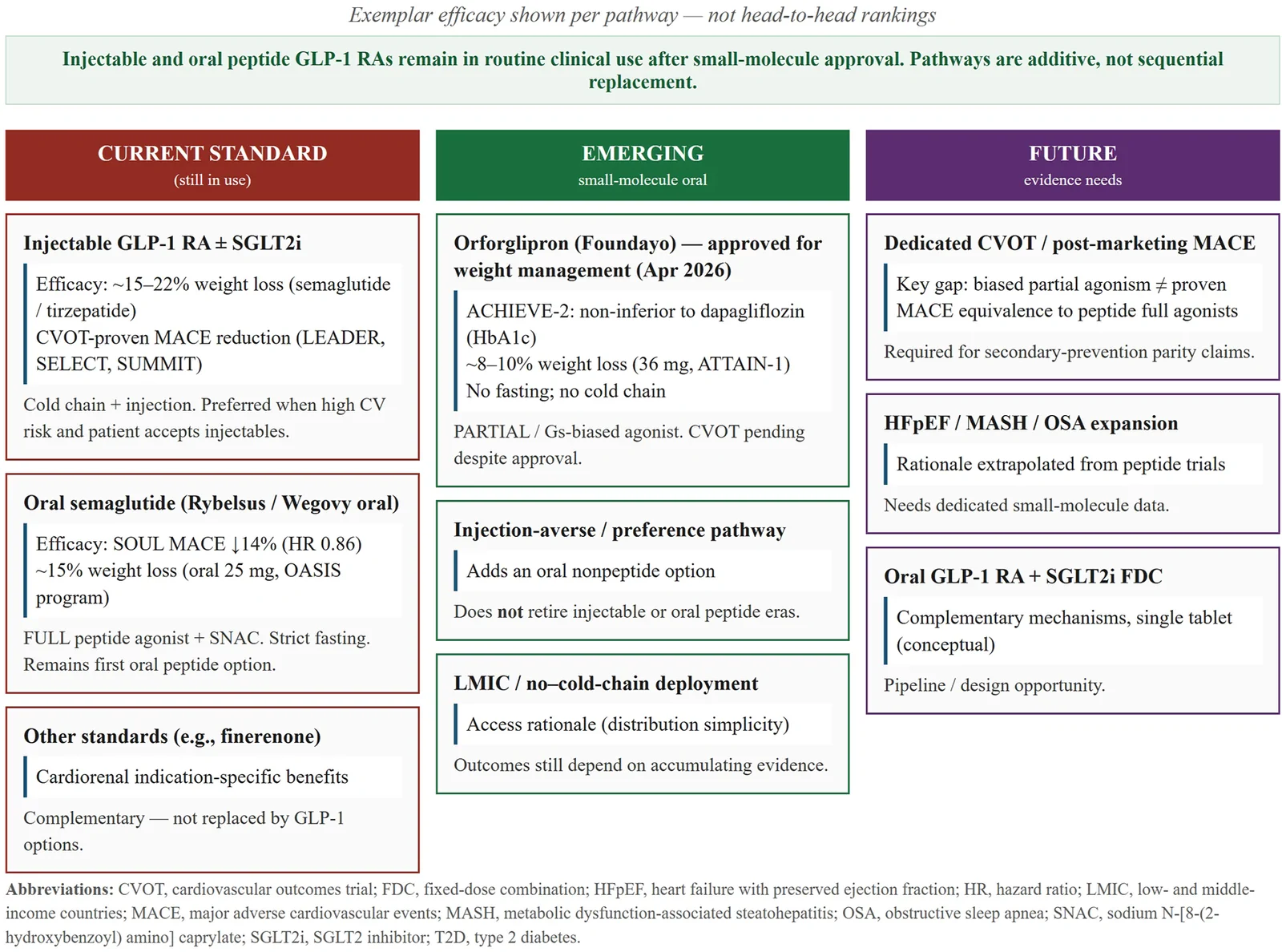
Cardiometabolic positioning with efficacy anchors. Positioning of oral small-molecule GLP-1 RAs in the cardiometabolic treatment algorithm. Three-column framework showing (left) current CVOT-proven standards with exemplar efficacy (injectable GLP-1 RAs; oral semaglutide/SOUL), (middle) emerging roles for orforglipron after weight-management approval (ACHIEVE glycemic data; no fasting/cold chain), and (right) future evidence needs (dedicated CVOT/post-marketing MACE, HFpEF/MASH/OSA, combinations). Earlier peptide therapies remain in routine use alongside newer oral options. Abbreviations: ASCVD, atherosclerotic cardiovascular disease; CKD, chronic kidney disease; CVOT, cardiovascular outcomes trial; FDC, fixed-dose combination; HFpEF, heart failure with preserved ejection fraction; LMIC, low- and middle-income countries; MACE, major adverse cardiovascular events; MASH, metabolic dysfunction-associated steatohepatitis; OSA, obstructive sleep apnea; SGLT2i, sodium-glucose cotransporter-2 inhibitor; T2D, type 2 diabetes.

ACHIEVE-2 provides the only direct head-to-head comparison between an oral small-molecule GLP-1 RA and an SGLT2 inhibitor (dapagliflozin) (Welch et al., 2026). Orforglipron 36 mg was non-inferior to dapagliflozin for HbA1c reduction and superior for weight loss. In clinical practice, the two classes are increasingly viewed as complementary rather than competitive, with the combination of SGLT2 inhibitor plus GLP-1 RA conferring additive benefits on glycemic control, weight, blood pressure, and cardiorenal outcomes. The comparative positioning of these oral GLP-1 RA strategies within the broader cardiometabolic treatment landscape is summarized in Figure 2, with a pharmacology–efficacy synopsis in Table 1.

## 5 Regulatory and access landscape

### 5.1 United States

Orforglipron (Foundayo; Eli Lilly) received FDA approval on 1 April 2026 (NDA 220934) as an adjunct to diet and physical activity for chronic weight management in adults with obesity, or overweight with at least one weight-related comorbidity (FDA NDA 220934, 2026). Approval under the Commissioner’s National Priority Voucher pathway does not eliminate the need for long-term cardiovascular and hepatic surveillance; the approval letter requires post-marketing evaluation of MACE and drug-induced liver injury, among other commitments. A separate type 2 diabetes indication remains under active clinical/regulatory development supported by the ACHIEVE program (Welch et al., 2026; Giorgino et al., 2026).

The FDA’s December 2025 approval of Wegovy (semaglutide) oral tablets (NDA 218316) for chronic weight management and cardiovascular risk reduction established a regulatory precedent for oral peptide GLP-1 RA therapy in cardiometabolic indications (FDA NDA 218316, 2025). Public FDA listings have also described tentative approvals for generic semaglutide injection and generic finerenone in 2026 (FDA ANDA 220314, 2026; FDA ANDA 220747, 2026); as originator peptide products face increasing generic competition, the incremental value of oral small-molecule agents will rest more on convenience, adherence, and preference than on cost alone.

### 5.2 Global perspective

In China, clinical trial sites participated in ACHIEVE-5, and domestic developers are advancing oral incretin programs. For low- and middle-income countries, small-molecule GLP-1 RAs offer a compelling access rationale: elimination of cold-chain requirements simplifies distribution, and once-daily oral dosing without fasting restrictions aligns with community-based care models—though affordability and outcomes evidence will determine real-world uptake.

### 5.3 Health economics

Formal cost-effectiveness analyses for orforglipron are not yet available. A 2026 budget-impact analysis of semaglutide in HFpEF from the German statutory health insurance perspective reported an ICER of $30,443/QALY over 5 years, suggesting that GLP-1 RAs for HFpEF may be cost-effective at conventional willingness-to-pay thresholds when cardiovascular benefits are incorporated (Estler et al., 2026).

## 6 Future directions and unanswered questions

### 6.1 The imperative for cardiovascular outcomes trials

Despite FDA approval of orforglipron for weight management, the single most critical *efficacy* gap for cardiometabolic positioning remains the absence of a completed, event-driven CVOT demonstrating MACE reduction comparable to peptide GLP-1 RAs. Injectable agents earned cardiovascular indications through LEADER, SUSTAIN-6, REWIND, and SELECT; oral peptide semaglutide did so through SOUL (Marso et al., 2016a; Marso et al., 2016b; Lincoff et al., 2023; McGuire et al., 2025). Orforglipron’s biased partial agonism creates a specific mechanistic uncertainty: if cardioprotection depends partly on signaling axes that are attenuated when β-arrestin recruitment is reduced, glycemic and weight efficacy could dissociate from hard cardiovascular endpoints. Conversely, if weight loss, blood-pressure lowering, and Gs-cAMP–dependent metabolic effects dominate, cardiovascular benefit may still accrue. Neither hypothesis is settled by surrogate biomarkers. Post-marketing MACE evaluation required at approval is necessary but not a substitute for adequately powered comparative cardiovascular evidence when positioning these agents against CVOT-proven peptide therapies in secondary prevention.

### 6.2 Heart failure with preserved ejection fraction

The SUMMIT trial established tirzepatide as the first agent to reduce cardiovascular death or worsening heart failure events in patients with HFpEF and obesity (Packer et al., 2025). STEP-HFpEF demonstrated that semaglutide 2.4 mg improves symptoms, functional capacity, and quality of life in this population (Kosiborod et al., 2023). The mechanistic rationale for benefit in HFpEF—weight reduction, afterload reduction, anti-inflammatory effects, improved endothelial function, and favorable modulation of epicardial adipose tissue—applies equally to small-molecule agonists (Beghini et al., 2025; Senapati and Mukherjee, 2026). A dedicated HFpEF outcomes trial for orforglipron would be a logical extension of the development program (Roy et al., 2026).

### 6.3 Metabolic dysfunction-associated steatohepatitis (MASH)

Injectable semaglutide 2.4 mg has expanded the therapeutic conversation around metabolic dysfunction–associated steatohepatitis (MASH) through dedicated development and regulatory pathways for liver disease; oral small-molecule agonists are attractive candidates for analogous programs given hepatic safety signals in pooled Phase 3 analyses (Wharton et al., 2026) and the convenience of oral dosing. Dedicated MASH trials of orforglipron would be required before any indication-specific claims.

### 6.4 Combination strategies

A fixed-dose combination tablet pairing an oral small-molecule GLP-1 RA with an SGLT2 inhibitor has substantial conceptual appeal. Both classes are orally bioavailable with complementary mechanisms. Such a combination could simplify polypharmacy while maximizing cardiometabolic protection.

### 6.5 Real-world effectiveness

Key post-marketing questions include: Does elimination of injection barriers translate into meaningfully higher real-world persistence? Will oral small-molecule GLP-1 RAs reduce disparities in access? Can these agents be safely initiated in primary care? Long-term pharmacovigilance for rare adverse events (medullary thyroid carcinoma, pancreatitis) is essential.

## 7 Conclusion

Oral small-molecule GLP-1 receptor agonists represent a genuine inflection point in incretin-based therapy. By eliminating the peptide backbone—and with it injection, cold-chain, and fasting-condition constraints—agents such as orforglipron expand access to a class that has transformed cardiometabolic care. The Phase 3 ACHIEVE and ATTAIN programs, pooled hepatic safety data exceeding 11,000 participants, and April 2026 FDA approval of Foundayo for chronic weight management establish a tangible clinical foothold.

However, evidence maturity is not parity with injectable or oral peptide GLP-1 RAs for cardiovascular risk reduction. Biased partial agonism provides a mechanistic reason to withhold assumptions of MACE equivalence until dedicated cardiovascular programs report. Indirect comparisons with oral semaglutide should be interpreted cautiously and not as head-to-head proof of superiority. Earlier eras of GLP-1 RA therapy (injectable and SNAC-enhanced oral peptides) remain fully in clinical use; small-molecule agents are additive options, not wholesale replacements.

The next phase of development—cardiovascular outcomes, T2D labeling maturation, real-world persistence, and equitable global deployment—will determine whether oral small-molecule GLP-1 RAs become foundational cardiometabolic therapies or remain convenience-focused alternatives.
